# Supplementary material for: State-dependent representations of mixtures by the olfactory bulb
Source: eLife. 2022 Mar 7;11:e76882. doi: 10.7554/eLife.76882 (PMC8937304; doi:10.7554/eLife.76882)
Supplement: Transparent reporting form [file elife-76882-transrepform1.pdf]

Westbrook Centre
Milton Road
Cambridge CB4 1YG, UK

P 01223 855340
W elifesciences.org
T @elife

eLife's transparent reporting form

We encourage authors to provide detailed information *within their submission* to facilitate the interpretation and replication of experiments. Authors can upload supporting documentation to indicate the use of appropriate reporting guidelines for health-related research (see EQUATOR Network), life science research (see the BioSharing Information Resource), or the ARRIVE guidelines for reporting work involving animal research. Where applicable, authors should refer to any relevant reporting standards documents in this form.

If you have any questions, please consult our Journal Policies and/or contact us: editorial@elifesciences.org.

Sample-size estimation

- You should state whether an appropriate sample size was computed when the study was being designed
- You should state the statistical method of sample size computation and any required assumptions
- If no explicit power analysis was used, you should describe how you decided what sample (replicate) size (number) to use

Please outline where this information can be found within the submission (e.g., sections or figure legends), or explain why this information doesn't apply to your submission:

> We based our sample sizes on the expected effect size (deviation from linear sum, measured by the absolute value of the z-score*) using anaesthetised mice (our data confirming previously published results). Cohen's D for this dataset was ~0.5. For α = 0.05 and β = 0.8. the minimum sample size for independent samples t-test was 64. However, for awake mice, the data was likely more variable, so we used substantially larger sample sizes (at least 179 ROIs for each of the three datasets from awake mice). Cohen's D was based on Cohen, J. (1988) Statistical Power Analysis for the Behavioural Sciences, 2nd ed. New York
>
> * the absolute difference between observed sum vs. linear sum, divided by the sum of standard deviations (standard deviations for the component odour responses and the observed mixtures)

Replicates

- You should report how often each experiment was performed
- You should include a definition of biological versus technical replication
- The data obtained should be provided and sufficient information should be provided to indicate the number of independent biological and/or technical replicates
- If you encountered any outliers, you should describe how these were handled
- Criteria for exclusion/inclusion of data should be clearly stated
- High-throughput sequence data should be uploaded before submission, with a private link for reviewers provided (these are available from both GEO and ArrayExpress)

Please outline where this information can be found within the submission (e.g., sections or figure legends), or explain why this information doesn't apply to your submission:

eLife Sciences Publications, Ltd is a limited liability non-profit non-stock corporation incorporated in the State of Delaware, USA, with company number 5030732, and is registered in the UK with company number FC030576 and branch number BR015634 at the address Westbrook Centre, Milton Road, Cambridge CB4 1YG.

Replicates for each condition are as follows:
- naïve, anaesthetised mice (glomerular imaging), 4 fields of view, 4 mice (line 483)
- naïve anaesthetised mice (somata imaging), 7 fields of view, 4 mice (line 503)
- Behavioural training, 7 mice (line 517)
- trained awake mice (somata imaging), 13 fields of view, 6 mice (line2 527-528)
- trained, anaesthetised mice (somata imaging), 8 fields of view, 4 mice (line 528)
- awake, engaged (random association) mice, 20 fields of view, 6 mice (line 561)
- awake, disengaged mice, 14 fields of view, 4 mice (line 562)

Data from sessions with motion artefacts or drifts were removed from analyses (lines 433-434). They were determined by by visual inspection based on frame averages (i.e., 400 frames per trial were averaged, and the averaged frames over time were inspected), as well as overlaying the ROIs on these averaged frames. Otherwise all data were included.

eLife Sciences Publications, Ltd is a limited liability non-profit non-stock corporation incorporated in the State of Delaware, USA, with company number 5030732, and is registered in the UK with company number FC030576 and branch number BR015634 at the address Westbrook Centre, Milton Road, Cambridge CB4 1YG.

Statistical reporting

- Statistical analysis methods should be described and justified
- Raw data should be presented in figures whenever informative to do so (typically when N per group is less than 10)
- For each experiment, you should identify the statistical tests used, exact values of N, definitions of center, methods of multiple test correction, and dispersion and precision measures (e.g., mean, median, SD, SEM, confidence intervals; and, for the major substantive results, a measure of effect size (e.g., Pearson's r, Cohen's d)
- Report exact p-values wherever possible alongside the summary statistics and 95% confidence intervals. These should be reported for all key questions and not only when the p-value is less than 0.05.

Please outline where this information can be found within the submission (e.g., sections or figure legends), or explain why this information doesn't apply to your submission:

List of tests with relevant details:
(1) ANOVA on lick preference index, n = 7 mice (lines 138-139; individual points shown in figure 3E)
(2) Two-sample K-S test on the fractional deviation distributions (lines 155-156)
(3) Mann-Whitney U test for comparison of SVM performances (lines 191-192)
(4) Mann-Whitney U test for comparison of SVM performances (lines 196-197)
(5) Paired t-test for behavioural performance with vs. without muscimol (lines 624-625)
(6) Paired t-test on the effect of sublinearity (Fig. 7E legends)

(For large datasets, or papers with a very large number of statistical tests, you may upload a single table file with tests, Ns, etc., with reference to sections in the manuscript.)

Group allocation

- Indicate how samples were allocated into experimental groups (in the case of clinical studies, please specify allocation to treatment method); if randomization was used, please also state if restricted randomization was applied
- Indicate if masking was used during group allocation, data collection and/or data analysis

Please outline where this information can be found within the submission (e.g., sections or figure legends), or explain why this information doesn't apply to your submission:

The experiments were not done blindly, since the stimulus-reward contingency was visible to the experimenter. However, the olfactometer performance, age and sex of the mice, and analyses codes used were the same for all conditions. (Lines 447-449)

Additional data files ("source data")

- We encourage you to upload relevant additional data files, such as numerical data that are represented as a graph in a figure, or as a summary table
- Where provided, these should be in the most useful format, and they can be uploaded as "Source data" files linked to a main figure or table
- Include model definition files including the full list of parameters used
- Include code used for data analysis (e.g., R, MatLab)
- Avoid stating that data files are "available upon request"

Please indicate the figures or tables for which source data files have been provided:

eLife Sciences Publications, Ltd is a limited liability non-profit non-stock corporation incorporated in the State of Delaware, USA, with company number 5030732, and is registered in the UK with company number FC030576 and branch number BR015634 at the address Westbrook Centre, Milton Road, Cambridge CB4 1YG.

Source data for comparing the (EB + MB) mixture responses for anaesthetised, trained and behaving, awake engaged and awake disengaged mice have been uploaded to Dryad. This corresponds to the time window 300-1000 ms after odour onset.

eLife Sciences Publications, Ltd is a limited liability non-profit non-stock corporation incorporated in the State of Delaware, USA, with company number 5030732, and is registered in the UK with company number FC030576 and branch number BR015634 at the address Westbrook Centre, Milton Road, Cambridge CB4 1YG.
